# Tween 80™-induced changes in fatty acid profile of selected mesophilic lactobacilli

**DOI:** 10.3389/abp.2024.13014

**Published:** 2024-07-04

**Authors:** Dorota Zaręba, Małgorzata Ziarno

**Affiliations:** ^1^ Professor E. Pijanowski Catering School Complex in Warsaw, Warsaw, Poland; ^2^ Institute of Food Science, Department of Food Technology and Assessment, Warsaw University of Life Sciences - SGGW (WULS-SGGW), Warsaw, Poland

**Keywords:** lactic acid bacteria, lactobacilli, fatty acid profile, functionality, Tween 80^TM^

## Abstract

Fatty acid profiles are crucial for the functionality and viability of lactobacilli used in food applications. Tween 80™, a common culture media additive, is known to influence bacterial growth and composition. This study investigated how Tween 80™ supplementation impacts the fatty acid profiles of six mesophilic lactobacilli strains (*Lacticaseibacillus* spp., *Limosilactobacillus* spp., *Lactiplantibacillus plantarum*). Analysis of eleven strains revealed 29 distinct fatty acids. Tween 80™ supplementation significantly altered their fatty acid composition. Notably, there was a shift towards saturated fatty acids and changes within the unsaturated fatty acid profile. While some unsaturated fatty acids decreased, there was a concurrent rise in cyclic derivatives like lactobacillic acid (derived from vaccenic acid) and dihydrosterculic acid (derived from oleic acid). This suggests that despite the presence of Tween 80™ as an oleic acid source, lactobacilli prioritize the synthesis of these cyclic derivatives from precursor unsaturated fatty acids. Myristic acid and dihydrosterculic acid levels varied across strains. Interestingly, palmitic acid content increased, potentially reflecting enhanced incorporation of oleic acid from Tween 80™ into membranes. Conversely, cis-vaccenic acid levels consistently decreased across all strains. The observed fatty acid profiles differed from previous studies, likely due to a combination of factors including strain-specific variations and growth condition differences (media type, temperature, harvesting point). However, this study highlights the consistent impact of Tween 80™ on the fatty acid composition of lactobacilli, regardless of these variations. In conclusion, Tween 80™ significantly alters fatty acid profiles, influencing saturation levels and specific fatty acid proportions. This work reveals key factors, including stimulated synthesis of lactobacillic acid, competition for oleic acid incorporation, and strain-specific responses to myristic and dihydrosterculic acids. The consistent reduction in cis-vaccenic acid and the presence of cyclic derivatives warrant further investigation to elucidate their roles in response to Tween 80™ supplementation.

## Introduction

The fatty acid composition of bacterial cells of the lactobacilli determines their survival and depends on many environmental factors (Johnsson et al., 1995; Partanen et al., 2001; Corcoran et al., 2007; Montanari et al., 2010; Li et al., 2011; Tan et al., 2012; Terraf et al., 2012; Al-Naseri et al., 2013; Hansen et al., 2015; Reitermayer et al., 2018). Corcoran et al. (2007) proved this by showing that oleic acid (C18:1,*cis*-9) and *cis-*vaccenic acid (C18:1,*cis*-11) have a protective effect on *Lacticaseibacillus rhamnosus* GG cells suspended in artificial gastric juice. These researchers have shown that only in the case of these two fatty acids, the number of bacterial cells was greater than the number of cells in the control sample. Other tested fatty acids, including stearic (C18:0), elaidic (C18:1,*trans*-9), linoleic (C18:2,*cis*-9,*cis*-12), *cis-*9,*trans-*11-octadecadienoic, and *trans-*10,*cis-*12-octadecadienoic, caused a decrease in the number of viable cells (to a level of 3.89 log CFU/mL in case of *trans-*10,*cis-*12-octadecadienoic acid and to 4.75 log CFU/mL for stearic and elaidic acids). This phenomenon can be explained by the fact that oleic (C18:1,*cis*-9) and *cis-*vaccenic (C18:1,*cis*-11) acids are the substrates for the synthesis of fatty acid in various ways required for cell survival and modification of cell membrane fluidity. Lactic acid bacteria (LAB), lactobacilli including, are equipped with the mechanisms for transforming these fatty acids in their cyclic, polyunsaturated, or conjugated forms (Henderson and McNeill, 1966; Weeks and Wakil, 1970; Partanen et al., 2001; Corcoran et al., 2007; Tan et al., 2012). Transformation of linoleic (C18:2,*cis*-9,*cis*-12) or conjugated acids to oleic acid (C18:1,cis-9) requires the action of specific enzymes if LAB cells lack access to external sources of oleic acid. In the absence of external oleic acid, some LAB strains may resort to this conversion pathway to maintain a certain level of oleic acid, potentially leading to a decrease in the overall protective action of these polyunsaturated fatty acids.

One of the external sources of fatty acids for bacterial cells is their living environment, including culture media. A key example is oleic acid, a fatty acid found in many natural sources. However, bacteria can also utilize oleic acid provided in the form of Tween 80™. Tween 80™ is a nonionic surfactant, commonly used in the food industry as an emulsifier, dispersant, and stabilizer. It finds application in microbiology as a common additive to media for cultivating lactobacilli (Johnsson et al., 1995; Partanen et al., 2001; Montanari et al., 2010; Li et al., 2018). It could be also employed in food fermentation processes for its beneficial effects on microbial growth and product quality (Li et al., 2018). Chemically, Tween 80™ is a synthetic molecule derived from sorbitan (a cyclic polyol) and esterified with oleic acid (C18:1,*cis*-9). This combination results in a molecule with a hydrophilic head (sorbitan) and a hydrophobic tail (oleic acid), allowing Tween 80™ to act as a surface-active agent. Tween 80™ improves the solubility and bioavailability of nutrients, promoting microbial growth and fermentation efficiency. By facilitating nutrient uptake and metabolism, Tween 80™ can lead to higher yields of fermented products (Li et al., 2018). The combination of oleic acid (C18:1,*cis*-9) with sorbitol permits the solubility of fatty acid in aqueous solution and a uniform dispersion of particles in growth medium, thereby making it more accessible to acid bacterial cells. Corcoran et al. (2007) compared the effect of adding Tween 80™ to the survival of four strains of lactobacilli suspended in artificial gastric juice: *Lacticaseibacillus rhamnosus* GG, *Lacticaseibacillus rhamnosus* E800, *Lacticaseibacillus paracasei* subsp. *paracasei* NFBC 338, and *Ligilactobacillus salivarius* UCC 500. In each case they confirmed the protective effect of Tween 80™ on cell viability. No Tween 80™ resulted in a decrease in the number of LAB viable cells in at least two logarithmic cycles after incubation in artificial gastric juice for 90 min. The cited studies are confirmed by other references (Johnsson et al., 1995; Broadbent et al., 2014).

The most widely used methods in study cellular fatty acid (as methyl esters) of Firmicutes (including lactobacilli) is matrix-assisted laser desorption/ionization time-of-flight mass spectrometry (MALDI-TOF) based on the principle of ionization and mass analysis of molecules, and it is particularly well-suited for identifying bacteria because it can analyze intact bacterial cells rather than extracting and purifying specific molecules (Anderson et al., 2014; Foschi et al., 2017; Nacef et al., 2017). As a result of these advantages, MALDI-TOF is becoming the preferred method for the identification of lactobacilli in many laboratories. MALDI-TOF (matrix-assisted laser desorption ionization time-of-flight mass spectrometry) is another method that is increasingly being used for the study of bacterial cellular fatty acid. It is a rapid, sensitive, and easy-to-use technique that can provide accurate species identification. However, the Sherlock Microbial Identification System (by MIDI Inc.) is a reliable method for species identification, particularly for lactobacilli, and is often used in conjunction with other methods, such as sequencing, to provide a complete taxonomic profile (Brondz, 2002; Kunitsky et al., 2006; Alexander, 2012). The alternative method referred to in the statement is likely to be gas chromatography-mass spectrometry (GC-MS), which is a more sophisticated technique that can provide more detailed information about the composition of fatty acids. However, GC-MS is a more expensive and time-consuming method, so it is not always the most practical choice. MIDI is a faster and more cost-effective method, and it is often sufficient for routine identification of lactobacilli. In general, MIDI is a suitable choice for routine identification of lactobacilli when speed and cost are important, while MALDI-TOF is the more advanced method and is the best choice for routine identification and specialized applications. GC-MS is most appropriate for more detailed studies or when species identification is challenging.

This study investigates the influence of Tween 80™ (oleic acid source) supplementation in growth media on the FA profile of selected mesophilic lactobacilli strains. We aim to compare their abilities to modulate FA synthesis and transformation upon incorporation of this external oleic acid (C18:1,*cis*-9) source.

## Materials and methods

### Materials

Eleven lactobacilli strains of the lactobacilli (Table 1) deposited in the laboratory collection in the form of monocultures freeze-dried or frozen were used in our research. Cultures of each bacterial strain were conducted in three independent experiments. First, the cultures were revived in the MRS (de Mann, Rogosa, and Sharpe) broth supplemented with Tween 80™ (Sigma-Aldrich) in portions of 50 mL (incubation at 37°C for approx. 12 h). Tween 80™ is known to influence membrane fluidity and potentially impact fatty acid synthesis in lactobacilli (Al-Naseri et al., 2013; Zotta et al., 2017b; Reitermayer et al., 2018). Subsequently, the cultures were inoculated onto the surface of three parallel MRS agar plates with Tween 80™ and three parallel MRS agar plates without Tween 80™ (in two independent replicates for each growth medium). Cultures were incubated at 37°C for 24 h under anaerobic conditions. Anaerocult™ A (Merck) was used to produce an anaerobic milieu in the anaerobic jar™ (Merck). Anaerocult™ A is recommended for the cultivation of obligatory and facultative anaerobes. To minimize growth phase variation, we carefully harvested cells for biomass collection from the defined center sector of each plate using the secondary inoculum. This ensured a representative sample of the cultured population for fatty acid analysis (Asadi et al., 2015).

**TABLE 1:** The collection of lactobacilli strains used in this study.

Species and strain (the origin)	Abbreviation
*Lacticaseibacillus rhamnosus* GG (ATCC 53103; American Type Culture Collection, United States)	Rh-GG
*Lacticaseibacillus rhamnosus* MB (Mediterranea Biotechnologies, Italy)	Rh-MB
*Lacticaseibacillus rhamnosus* ATCC 7469 (American Type Culture Collection, United States)	Rh-74
*Lacticaseibacillus casei* ATCC 393 (American Type Culture Collection, United States)	C-393
*Lacticaseibacillus casei* 431 (Chr. Hansen, Denmark)	C-431
*Lacticaseibacillus paracasei* subsp. *paracasei* MB (Mediterranea Biotechnologies, Italy)	PP
*Limosilactobacillus fermentum* ATCC 9338 (American Type Culture Collection, United States)	F-36
*Limosilactobacillus reuteri* DSM 17938 (Leibniz Institute DSMZ - German Collection of Microorganisms and Cell Cultures, Germany)	Re-P
*Lactiplantibacillus plantarum* 299v (DSM 9843; Leibniz Institute DSMZ-German Collection of Microorganisms and Cell Cultures, Germany)	P-299
*Lactiplantibacillus plantarum* NCAIM B.01149 (The National Collection of Agricultural and Industrial Microorganisms, Corvinus University of Budapest, Hungary)	P-011
*Lactiplantibacillus plantarum* NCAIM B.01834 (The National Collection of Agricultural and Industrial Microorganisms, Corvinus University of Budapest, Hungary)	P-018

### The extraction of fatty acids: separating and detecting conditions of fatty acid methyl esters using GC-MS

The fatty acids were extracted from the entire bacterial biomass according to recommendations (Haack et al., 1994; Buyer, 2002; Kankaanpaa et al., 2004). Each bacterial biomass sample was prepared for analysis and analyzed in two replicates. The chromatographic separation of fatty acid methyl esters was carried out by gas chromatography coupled with mass spectrometer (GC-MS QP 2010 Shimadzu, Shimadzu Corporation, Japan) using polar column 007-23-30-0.2F (30 m × 0.25 mm × 0.20 μm; Quadrex, Quadrex Corporation, United States). The sample was injected under a split ratio of 1:25 at the dispenser temperature equal to 230°C. The chromatographic separation was conducted in the following conditions: Initial temperature of column of 60°C, 2 min isotherm, the temperature increase of 4°C/min to 220°C, 10 min isotherm. The carrier gas was helium with a flow rate of 0.37 mL/min. The following detector conditions were used: Temperature of the ion source of 200°C, temperature of the line connecting GC with MS of 220°C, detector voltage of 1.45 kV, and quadrupole filter sweep in a range 50-400 m/z. The following standards were used for identifying fatty acids: oleic acid (Sigma Aldrich, United States), anteiso12-methyltetradecanoic acid (Sigma Aldrich, United States), 2-hydroxytetradecanoic acid (Sigma Aldrich, United States), nonadecanoic acid (Sigma Aldrich, United States), the BAME (bacterial acid methyl esters; Sigma Aldrich, United States), GLC-674 and GLC-617 (Nu-Chek-Prep., United States), and isomers of methyl esters of linoleic acid 18:2 (*cis-*9,*trans-*11 and *trans-*10,*cis-*12; Nu-Chek-Prep., United States). The composition of bacterial acid methyl esters (BAMEs) is presented in Supplementary Table 1S. In other cases of fatty acids (including lactobacillic acid), the comparisons were made with the literature data (Brondz, 2002; Christie et al., 2007; Montanari et al., 2010). The data obtained from GC-MS was analyzed using GCMS Solution v.2.50 (Shimadzu Corporation).

### Statistical analysis

The statistical analysis was carried out using the statistical program Statistica v.10. The analysis of variance (ANOVA) was employed. In this study, ANOVA was complemented by Tukey’s test, conducted at a significance level of 0.05, to scrutinize the mutual influence of the factors and identify any statistically significant differences between them. Principal components analysis (PCA) was conducted to assess the relationship between fatty acid profiles and the presence or absence of Tween 80™ in the growth medium. In this case, PCA was used to identify the main components that influenced the variation in fatty acid profiles.

## Results and discussion

### Lacticaseibacillus rhamnosus

The determined fatty acid profile of *L. rhamnosus* GG, *L. rhamnosus* MB, and *L. rhamnosus* ATCC 4769 cells demonstrated the presence of 29 fatty acids after the incubation on the growth medium with Tween 80™ (Table 2). The strains of *L. rhamnosus* used in our study were characterized by an intense growth on the MRS agar without Tween 80™. The character of the growth of this species’ colonies was different from other lactobacilli species used for this study. The colonies on the line of loop movement were snow-white with characteristic papules and damming, and so obtaining the biomass required for the analysis was not difficult. Nineteen fatty acids were identified in the fatty acid profile of *L. rhamnosus* GG cells, while the presence of 21 fatty acids was noted in the case of *L. rhamnosus* MB and *L. rhamnosus* ATCC 7469 cells (Table 2).

**TABLE 2:** The percentage fatty acids composition (mean ± SD, n = 6) in mesophilic *Lacticaseibacillus rhamnosus* strains profiles obtained from the cell cultured in a growth medium with Tween 80^TM^ (with T80) and without Tween 80^TM^ (no T80).

Strain symbol	*L. rhamnosus* GG		*L. rhamnosus* MB		*L. rhamnosus* ATCC 7469		*p-value*
Fatty acid/Medium	With T80	No T80	With T80	No T80	With T80	No T80	
C10:0	0.09^a^ ± 0.06	0.10^a^ ± 0.07	0.10^a^ ± 0.04	0.05^a^ ± 0.06	0.16^a^ ± 0.13	0.14^a^ ± 0.04	0.5612
C12:0	0.50^b^ ± 0.09	0.21^a,b^ ± 0.13	0.58^b^ ± 0.04	0.06^a^ ± 0.02	1.03^b,c^ ± 0.64	0.23^a,b^ ± 0.09	0.0108
C14:0	6.62^c^ ± 0.79	4.58^b^ ± 1.27	4.50^b^ ± 0.30	2.83^a^ ± 0.19	2.86^a^ ± 0.70	2.23^a^ ± 0.49	0.0001
15:0,*iso*	0.18^a,b^ ± 0.02	0.33^b,c^ ± 0.10	0.12^a^ ± 0.01	0.22^a^ ± 0.01	0.16^a^ ± 0.06	0.25^a,b^ ± 0.03	0.0036
15:0,*anteiso*	0.03^a^ ± 0.03	0.00^a^ ± 0.00	0.08^a^ ± 0.02	0.02^a^ ± 0.02	0.05^a^ ± 0.05	0.03^a^ ± 0.05	0.1106
C15:0	0.04^b^ ± 0.02	0.00^a^ ± 0.01	0.09^b^ ± 0.02	0.04^b^ ± 0.01	0.06^b^ ± 0.04	0.03^a,b^ ± 0.05	0.0336
C16:0	35.82^b^ ± 1.54	48.51^d^ ± 2.27	30.20^a^ ± 1.95	42.09^c^ ± 0.75	28.67^a^ ± 0.48	33.86^b^ ± 0.20	0.0001
C16:1,*trans-*9	1.04^a,b^ ± 0.11	1.93^c^ ± 0.49	0.76^a^ ± 0.09	1.88^b,c^ ± 0.11	1.13^b^ ± 0.10	2.03^c^ ± 0.08	0.0001
C16:1,*cis-*9	1.74^c^ ± 0.12	1.69^b,c^ ± 0.35	1.16^a,b^ ± 0.15	1.25^a,b^ ± 0.07	1.28^a,b^ ± 0.13	1.10^a^ ± 0.07	0.0523
C12:0,2OH	0.22^a^ ± 0.02	0.24^a^ ± 0.08	0.22^a^ ± 0.02	0.31^b^ ± 0.03	0.25^a^ ± 0.05	0.33^b^ ± 0.04	0.0430
*cyc*C17:0,*cis-*9,10	0.10^a,b^ ± 0.03	0.47^c^ ± 0.13	0.04^a^ ± 0.01	0.21^b^ ± 0.02	0.21^b^ ± 0.03	0.42^c^ ± 0.06	0.0001
C18:0	3.07^a,b^ ± 0.66	4.50^b^ ± 0.29	4.16^b^ ± 1.56	7.64^d^ ± 0.26	2.81^a^ ± 0.18	6.56^c^ ± 0.24	0.0001
C18:1	0.16^b^ ± 0.02	0.00^a^ ± 0.00	0.14^b^ ± 0.07	0.00^a^ ± 0.00	0.15^b^ ± 0.07	0.00^a^ ± 0.00	0.0003
C18:1,*trans-*6	0.12^a,b^ ± 0.02	0.25^c^ ± 0.06	0.07^a^ ± 0.01	0.17^b^ ± 0.02	0.17^b^ ± 0.03	0.34^d^ ± 0.05	0.0001
C18:1,*trans-*9	0.14^b^ ± 0.01	0.32^d^ ± 0.09	0.08^a^ ± 0.01	0.23^b,c^ ± 0.02	0.19^b^ ± 0.04	0.41^e^ ± 0.03	0.0001
C18:1,*trans-*11	0.25^c^ ± 0.08	0.00^a^ ± 0.00	0.49^d^ ± 0.06	0.06^a^ ± 0.09	0.19^b^ ± 0.08	0.00^a^ ± 0.00	0.0001
C18:1,*cis-*6	0.23^a^ ± 0.10	0.37^a,b^ ± 0.09	0.46^b^ ± 0.03	0.62^c^ ± 0.12	0.36^a,b^ ± 0.09	0.84^d^ ± 0.12	0.0001
C18:1,*cis-*9	10.03^c^ ± 0.72	0.38^a^ ± 0.30	13.87^d^ ± 1.03	0.72^a^ ± 0.09	9.21^b^ ± 0.26	0.15^a^ ± 0.04	0.0001
C18:1,*cis-*11	9.49^a^ ± 0.63	13.85^b^ ± 1.73	10.10^a^ ± 0.42	20.52^c^ ± 1.12	14.36^b^ ± 0.64	20.21^c^ ± 0.82	0.0001
C18:2,*trans-*9,*trans-*12	0.01^a^ ± 0.02	0.37^a^ ± 0.72	0.03^a^ ± 0.04	0.10^a^ ± 0.02	0.06^a^ ± 0.05	0.17^a^ ± 0.05	0.1889
C18:2,*cis-*9, *cis-*12	0.05^a,b^ ± 0.06	0.00^a^ ± 0.00	0.07^a,b^ ± 0.02	0.00^a^ ± 0.00	0.00^a^ ± 0.00	0.00^a^ ± 0.00	0.0075
*cyc*C19:0,*cis-*9,10	15.45^b^ ± 1.94	0.23^a^ ± 0.10	21.20^b,c^ ± 2.39	1.23^a^ ± 0.32	17.93^b^ ± 1.24	0.43^a^ ± 0.26	0.0001
*cyc*C19:0,*cis-*10,11	11.90^b^ ± 1.72	21.49^d,e^ ± 0.57	7.26^a^ ± 0.81	19.08^c^ ± 1.69	16.16^c^ ± 1.86	29.11^e^ ± 1.11	0.0001
18:2,*cis-*9,*trans-*11	0.92^b,c^ ± 0.06	0.20^a^ ± 0.04	1.43^c^ ± 0.12	0.68^b^ ± 0.04	1.30^c^ ± 0.17	1.15^b,c^ ± 0.13	0.0001
C18:2CLA_1	0.06^b^ ± 0.03	0.00^a^ ± 0.00	0.14^b^ ± 0.02	0.00^a^ ± 0.00	0.07^b^ ± 0.06	0.00^a^ ± 0.00	0.0003
18:2,*trans-*10,*cis-*12	0.82^b^ ± 0.05	0.00^a^ ± 0.00	1.16^b^ ± 0.11	0.00^a^ ± 0.00	0.48^b^ ± 0.07	0.00^a^ ± 0.00	0.0001
C18:2_CLA_2	0.09^b^ ± 0.01	0.00^a^ ± 0.00	0.14^b^ ± 0.01	0.00^a^ ± 0.00	0.06^a,b^ ± 0.05	0.00^a^ ± 0.00	0.0001
C18:2_CLA_3	0.05^b^ ± 0.02	0.00^a^ ± 0.00	0.15^b,c^ ± 0.01	0.00^a^ ± 0.00	0.03^a^ ± 0.03	0.00^a^ ± 0.00	0.0001
C18:2_CLA_4	0.76^b^ ± 0.09	0.00^a^ ± 0.00	1.20^c^ ± 0.13	0.00^a^ ± 0.00	0.61^b^ ± 0.19	0.00^a^ ± 0.00	0.0001
St/Lb	0.26^b^ ± 0.36	0.21^b^ ± 0.01	0.56^d^ ± 0.20	0.40^c^ ± 0.01	0.18^a^ ± 0.01	0.23^b^ ± 0.01	0.0001
O/V	1.06^c^ ± 0.01	0.03^a^ ± 0.02	1.37^d^ ± 0.01	0.04^a^ ± 0.01	0.64^b^ ± 0.01	0.01^a^ ± 0.00	0.0001
∑U	25.96^b^ ± 2.15	19.36^a^ ± 3.87	31.45^d^ ± 2.30	26.23^b^ ± 1.70	29.66^c^ ± 2.10	26.40^b^ ± 1.40	0.0009
∑S	74.02^a^ ± 6.28	80.66^a^ ± 5.02	68.55^a^ ± 7.20	73.78^a^ ± 3.40	70.36^a^ ± 5.50	73.62^a^ ± 2.70	0.1708
U/S	0.35^b^ ± 0.01	0.24^a^ ± 0.03	0.46^c^ ± 0.01	0.36^b^ ± 0.01	0.42^c^ ± 0.01	0.36^b^ ± 0.01	0.0001
∑CLA	2.70^d^ ± 0.26	0.20^a^ ± 0.04	4.22^e^ ± 0.40	0.68^b^ ± 0.01	2.55^d^ ± 0.60	1.15^c^ ± 0.10	0.0001

Legend: C10:0, caproic/decanoic acid; C12:0, lauric/dodecanoic acid; C14:0, myristic/tetradecanoic acid; C15:0,iso, iso-13-methyltetradecanoic acid; C15:0,anteiso, anteiso-12-methyltetradecanoic acid; C15:0, pentadecanoic acid; C16:0, palmitic/hexadecanoic acid; C16:1,*trans-*9, palmitelaidic/*trans-*9-hexadecenoic acid; C16:1,*cis-*9, palmitoleic/*cis-*9-hexadecenoic acid; C12:0,2OH, 2-hydroxydodecanoic acid; cycC17:0,*cis-*9, 10, *cis-*9, 10-methylenehexadecanoic acid; C18:0, stearic/octadecanoic acid; C18:1, octadecenoic acid; C18:1,*trans-*6, petroselaidic acid/*trans-*6-octadecenoic acid; C18:1,*trans-*9, elaidic acid/*trans-*9-octa-decenoic acid; C18:1,*trans-*11, *trans-*vaccenic acid/*trans-*11-octadecenoic acid; C18:1,*cis-*6, petroselinic acid/*cis-*6-octadecenoic acid; C18:1,*cis-*9, oleic/*cis-*9-octadecenoic acid; C18:1,*cis-*11, *cis-*vaccenic acid/*cis-*11-octadecenoic acid; C18:2,*trans-*9, *trans-*12, linoelaidic/*trans-*9, *trans-*12-octadecadienoic acid; C18:2,*cis-*9, *cis-*12, linoleic/*cis-*9, *cis-*12-octadecadienoic acid; cycC19:0,*cis-*9, 10, dihydrosterculic/*cis-*9, 10-methyleneoctadecanoic acid; cycC19:0,*cis-*10, 11, lactobacillic acid/*cis-*11, 12-methyleneoctadecanoic acid; 18:2,*cis-*9, *trans-*11, conjugated octadecadienoic acid; C18:2, conjugated octadecadienoic acid; 18:2_CLA_1 to _4, different isomers of CLA (their specific structures have not been determined); St/Lb, the ratio dihydrosterculic acid/lactobacillic acid; O/V, the ratio oleic acid/*cis-*vaccenic acid; S, saturated acids; U, unsaturated acids; U/S, the ratio unsaturated acids/saturated acids; ∑CLA, total sum of conjugated fatty acids.

^a,b,c,d,e^–Means with different lowercase letters the same line indicate a significant difference at the significance level of 0.05 (Tukey HSD).

By comparing the test strains of *L. rhamnosus* species acid, similarities were observed in the sequence fatty acid dominant in the fatty acid profile of the cells. The comparative analysis (conducted using Tukey’s test) of the share of all dominant fatty acids in the fatty acid profiles obtained from the biomass of all strains showed significant differences. The fatty acid profile of the *L. rhamnosus* GG strain was dominated (the share >10%) by palmitic (C16:0), dihydrosterculic (*cyc*C19:0,*cis*-9,10), lactobacillic (*cyc*C19:0,*cis*-10,11), and oleic (C18:1,*cis*-9,) fatty acids. Meanwhile, the fatty acid profile of the *L. rhamnosus* MB strain was dominated (the share >10%) by palmitic acid, dihydrosterculic acid (*cyc*C19:0,*cis*-9,10), oleic acid, and *cis*-vaccenic fatty (C18:1,*cis*-11) acids. As for the fatty acid profile of *L. rhamnosus* ATCC 7469 strain, it was dominated by palmitic (C16:0), dihydrosterculic (*cyc*C19:0,*cis*-9,10), lactobacillic (*cyc*C19:0,*cis*-10,11), and *cis*-vaccenic acid (C18:1,*cis*-11) acids (the share >10%). Their contribution to the fatty acid profile changed significantly when Tween 80™ was eliminated from the growth medium. The only fatty acids whose proportion in the fatty acid profile did not change significantly were the following fatty acids: caproic (C10:0), anteiso-12-methyltetradecanoic (C15:0,*anteiso*), palmitoleic (C16:1,*cis*-9), and linoelaidic (C18:2,*trans*-9,*trans*-12) fatty acids. In general, removal of Tween 80™ from the growth medium resulted in a decrease in the proportion of 14 fatty acids in the fatty acid profile, both saturated and unsaturated fatty acids, and an increase in the proportion of 11 fatty acids in the profile of fatty acids, mainly unsaturated fatty acids. Of course, these changes were not observed in every *L. rhamnosus* strains tested. Consequently, this resulted in a significant reduction in the calculated the ratio dihydrosterculic acid (*cyc*C19:0,*cis*-9,10)/lactobacillic acid (*cyc*C19:0,*cis*-10,11) (St/Lb ratio) with the exception for the *L. rhamnosus* MB strain, the ratio oleic acid (C18:1,*cis*-9)/*cis*-vaccenic acid (C18:1,*cis*-11) (O/V ratio), and the ratio unsaturated acids/saturated fatty acids (U/S ratio). It is worth noting that removal of Tween 80™ from the growth medium resulted in a significant reduction in the proportion of total unsaturated fatty acids, including conjugated fatty acids (CLA), in the fatty acid profile, but also did not result in a complete change in the proportion of saturated fatty acids in the fatty acid profile.

Kankaanpaa et al. (2004) found that *L. rhamnosus* GG cells grown with Tween 80™ contained oleic (21.5%), *cis*-vaccenic (21.8%), dihydrosterculic (23.5%), and lactobacillic (2.1%) acids as major fatty acids, along with conjugated acids at about 3%. Bacteria produce unsaturated fatty acids with various functionalities *de novo* (Zhu et al., 2019). These modifications, along with other fatty acids, increase the fluidity of the cytoplasmic membrane through their specific structures (Kaneda, 1991). The ability of the strains to synthesize *cis*-vaccenic acid and convert it to lactobacillic acid is noteworthy, with minimal *de novo* synthesis of oleic acid, especially in the absence of Tween 80™. The presence of Tween 80™ appears to inhibit the FAS system, as suggested by (Zhang et al., 2020). Our study indicates that all strains produced vaccenic acid even under anaerobic conditions, which is significant since anaerobic conditions favor the anaerobic vaccenic acid pathway. This contrasts with aerobic conditions in *L. casei* N87, which enhance growth, metabolism, and stress tolerance, influencing vaccenic acid synthesis (Ianniello et al., 2016). Refrigerated storage before biomass extraction can increase the proportion of unsaturated fatty acids (Zhu et al., 2019), which helps maintain membrane fluidity and protein activity (Santivarangkna et al., 2008; Parsons and Rock, 2013). The absence of oleic acid stimulates lactobacillic acid synthesis, while its presence increases dihydrosterculic acid levels (Kankaanpaa et al., 2004). This adaptive modification in fatty acid composition helps control membrane fluidity, aiding bacterial stress defense (Beal et al., 2001; Murga et al., 2001; Montanari et al., 2010; Pawar and Aranha, 2022). Environmental factors significantly influence unsaturated fatty acid proportions in lactobacilli membranes (Zotta et al., 2017a; Fonseca et al., 2019), with oxygen availability playing a crucial role (López de Felipe, 2023). Under aerobic conditions, lactobacilli can convert palmitoleic acid to cis-vaccenic acid (Cronan, 2002; Schujman and Mendoza, 2008; Jimenez et al., 2023). Corcoran et al. (2007) noted a rise in certain acids in *L. rhamnosus* GG cells when exposed to oleic acid (C18:1,*cis*-9). Our research found that adding oleic acid via Tween 80™ increased dihydrosterculic acid (*cyc*C19:0,*cis*-9,10), oleic acid (C18:1,*cis*-9), and conjugated fatty acids, while reducing palmitic (C16:0) and stearic (C18:0) acids. Furthermore, *L. rhamnosus* GG, *L. rhamnosus* MB, and *L. rhamnosus* ATCC 7469 cells cultured without Tween 80™ showed lactobacillic acid (*cyc*C19:0,*cis*-10,11) dominance in their fatty acid profiles. This difference is likely due to the lack of separation of cyclic acids in the Corcoran et al. (2007) study.

### *Lacticaseibacillus casei* and *Lacticaseibacillus paracasei*

The identified fatty acid profile of *L. casei* 393, *L. casei* 431, and *L. paracasei* subsp. *paracasei* MB grown on MRS agar with Tween 80™ contained 29 and 27 fatty acids for *L. casei* and *L. paracasei*, respectively (Table 3). The predominant fatty acids in the fatty acid profile of the first of these strains were: palmitic acid (C16:0), dihydrosterculic acid (*cyc*C19:0,*cis*-9,10), lactobacillic acid (*cyc*C19:0,*cis*-10,11), and *cis*-vaccenic acid (C18:1,*cis*-11). Meanwhile, fatty acids such as palmitic acid, oleic acid (C18:1,*cis*-9), *cis*-vaccenic acid (C18:1,*cis*-11), and dihydrosterculic acid (*cyc*C19:0,*cis*-9,10) were found to be dominant for the fatty acid profile of *L. casei* 431. On the other hand, dominant fatty acids in the *L. paracasei* subsp. paracasei MB fatty acid profile included oleic acid (C18:1,*cis*-9), dihydrosterculic acid (*cyc*C19:0,*cis*-9,10), and palmitic acid (C16:0). While dihydrosterculic acid (*cyc*C19:0,*cis*-9,10) could potentially be used to differentiate between *L. casei* and *L. paracasei* subsp. *paracasei* MB, the high proportion observed in this study might be due to stress experienced by the bacteria during growth (Rouch et al., 2002; Wu et al., 2020; Klein et al., 2022). Cyclic fatty acids, including dihydrosterculic acid (*cyc*C19:0,*cis*-9,10), are known markers of stress in LAB. Further studies are needed to investigate the influence of growth conditions on the fatty acid profile of *L. paracasei* subsp. *paracasei* MB and confirm its potential as a differentiating factor. *L. casei* and *L. paracasei* grown without Tween 80™ displayed altered fatty acid profiles compared to cultures with Tween 80™. Notably, the absence of oleic acid (C18:1,*cis*-9) triggered *cis*-vaccenic (C18:1,*cis*-11) and lactobacillic acid (*cyc*C19:0,*cis*-10,11) synthesis. Total saturated fatty acids, especially iso-13-methyltetradecanoic (C15:0,iso) and stearic (C18:0) acids, increased in bacteria lacking Tween 80™, although this effect was not universal. Conversely, unsaturated fatty acid content and the U/S ratio decreased without Tween 80™. Interestingly, total CLA and St/Lb and O/V ratios also significantly declined. These changes in *L. casei* and *paracasei* are distinct from those observed previously in*L. rhamnosus*.

**TABLE 3:** The percentage fatty acids composition (mean ± SD, n = 6) in mesophilic *Lacticaseibacillus casei* and *Lacticaseibacillus paracasei* strains profiles obtained from the cell cultured in a growth medium with Tween 80^TM^ (with T80) and without Tween 80^TM^ (no T80).

Strain symbol	*L. casei* ATCC 393		*L. casei* 431		*L. paracasei* subsp. *paracasei* MB		*p-value*
Fatty acid/Medium	With T80	No T80	With T80	No T80	With T80	No T80	
C10:0	0.16^c^ ± 0.01	0.10^c^ ± 0.03	0.01^a^ ± 0.03	0.03^a^ ± 0.08	0.05^a,b^ ± 0.03	0.05^a,b^ ± 0.04	0.0507
C12:0	0.70^b^ ± 0.08	0.10^a^ ± 0.03	0.80^b,c^ ± 0.09	0.18^a^ ± 0.08	1.94^c^ ± 0.37	0.24^a,b^ ± 0.08	0.0001
C14:0	6.29^c^ ± 0.38	3.19^a^ ± 0.10	7.92^d^ ± 0.35	4.72^a,b^ ± 0.21	6.32^c^ ± 0.38	3.24^a^ ± 0.70	0.0001
15:0,*iso*	0.52^b,c^ ± 0.04	0.63^c^ ± 0.03	0.70^c,d^ ± 0.06	0.88^d^ ± 0.03	0.12^a^ ± 0.02	0.40^b^ ± 0.06	0.0001
15:0,*anteiso*	0.06^a^ ± 0.02	0.00^a^ ± 0.00	0.07^a,b^ ± 0.04	0.02^a^ ± 0.05	0.13^a,b^ ± 0.03	0.05^a^ ± 0.04	0.0089
C15:0	0.06^a^ ± 0.02	0.00^a^ ± 0.00	0.09^a,b^ ± 0.05	0.05^a^ ± 0.03	0.17^b^ ± 0.02	0.10^b^ ± 0.03	0.0003
C16:0	19.81^a^ ± 0.60	22.82^b^ ± 0.28	24.36^b^ ± 2.87	35.62^c^ ± 0.40	20.81^a^ ± 1.99	42.38^d^ ± 1.57	0.0001
C16:1,*trans-*9	2.71^c^ ± 0.14	4.50^e^ ± 0.18	1.37^b^ ± 0.19	3.18^c,d^ ± 0.14	0.36^a^ ± 0.12	2.49^c^ ± 0.07	0.0001
C16:1,*cis-*9	4.61^b,c^ ± 0.22	3.89^b^ ± 0.23	5.87^c^ ± 0.26	7.11^d^ ± 0.29	1.29^a^ ± 0.06	2.59^a,b^ ± 0.36	0.0001
C12:0,2OH	0.42^b^ ± 0.01	0.51^c^ ± 0.01	0.51^c^ ± 0.05	0.84^d^ ± 0.04	0.21^a^ ± 0.03	0.44^b^ ± 0.04	0.0001
*cyc*C17:0,*cis-*9,10	0.39^c^ ± 0.03	0.67^d^ ± 0.06	0.02^a^ ± 0.02	0.17^b^ ± 0.02	0.08^a^ ± 0.06	0.17^b^ ± 0.03	0.0001
C18:0	1.02^a^ ± 0.77	0.95^a^ ± 0.08	0.90^a^ ± 0.12	1.11^a^ ± 0.21	1.77^a^ ± 0.22	14.27^b^ ± 0.89	0.0001
C18:1	0.17^b^ ± 0.04	0.00^a^ ± 0.00	0.07^a,b^ ± 0.05	0.00^a^ ± 0.00	0.22^b^ ± 0.06	0.00^a^ ± 0.00	0.0001
C18:1,*trans-*6	0.17^b,c^ ± 0.01	0.24^d^ ± 0.05	0.03^a,b^ ± 0.03	0.08^b^ ± 0.01	0.00^a^ ± 0.00	0.12^b^ ± 0.02	0.0001
C18:1,*trans-*9	0.20^c^ ± 0.02	0.32^d^ ± 0.04	0.04^a^ ± 0.01	0.13^b^ ± 0.02	0.00^a^ ± 0.00	0.12^b^ ± 0.02	0.0001
C18:1,*trans-*11	0.16^a^ ± 0.05	0.06^a^ ± 0.16	0.47^b^ ± 0.14	0.30^b^ ± 0.15	0.66^c^ ± 0.07	0.12^a^ ± 0.22	0.0018
C18:1,*cis-*6	0.25^a^ ± 0.04	0.46^b^ ± 0.05	0.26^a^ ± 0.06	0.36^a,b^ ± 0.08	0.20^a^ ± 0.05	0.52^b,c^ ± 0.05	0.0001
C18:1,*cis-*9	7.12^b^ ± 0.44	0.17^a^ ± 0.02	22.23^c^ ± 2.91	0.90^a^ ± 0.41	34.26^d^ ± 4.42	2.44^a,b^ ± 0.57	0.0001
C18:1,*cis-*11	16.19^b^ ± 0.33	23.84^d^ ± 0.80	15.28^b^ ± 1.78	30.76^e^ ± 0.93	4.96^a^ ± 1.38	18.69^c^ ± 2.25	0.0001
C18:2,*trans-*9,*trans-*12	0.33^b,c^ ± 0.02	0.55^c,d^ ± 0.06	0.23^b^ ± 0.04	0.49^c^ ± 0.08	0.06^a^ ± 0.04	0.25^b^ ± 0.05	0.0001
C18:2,*cis-*9, *cis-*12	0.03^a^ ± 0.01	0.00^a^ ± 0.00	0.07^a,b^ ± 0.04	0.00^a^ ± 0.00	0.08^a,b^ ± 0.03	0.02^a,b^ ± 0.04	0.0098
*cyc*C19:0,*cis-*9,10	19.23^d^ ± 1.43	0.64^a^ ± 0.12	11.36^c^ ± 1.53	0.55^a^ ± 0.19	20.89^d^ ± 1.33	3.29^b^ ± 1.15	0.0001
*cyc*C19:0,*cis-*10,11	18.00^e^ ± 0.75	35.83^f^ ± 0.83	3.76^b^ ± 0.58	12.09^d^ ± 0.58	1.17^a^ ± 0.86	7.21^c^ ± 0.81	0.0001
18:2,*cis-*9,*trans-*11	0.45^b^ ± 0.05	0.19^a^ ± 0.03	1.12^c^ ± 0.16	0.25^a,b^ ± 0.03	1.22^d^ ± 0.18	0.38^b^ ± 0.06	0.0001
C18:2CLA_1	0.04^a,b^ ± 0.02	0.00^a^ ± 0.00	0.13^c^ ± 0.03	0.00^a^ ± 0.00	0.16^c^ ± 0.03	0.00^a^ ± 0.00	0.0001
18:2,*trans-*10,*cis-*12	0.44^b^ ± 0.04	0.00^a^ ± 0.00	0.98^c^ ± 0.14	0.00^a^ ± 0.00	1.18^d^ ± 0.11	0.09^a^ ± 0.03	0.0001
C18:2_CLA_2	0.07^a^ ± 0.02	0.13^b^ ± 0.03	0.13^b^ ± 0.02	0.07^a^ ± 0.01	0.14^b^ ± 0.02	0.06^a^ ± 0.01	0.0004
C18:2_CLA_3	0.06^a^ ± 0.03	0.19^b^ ± 0.03	0.11^a,b^ ± 0.05	0.12^a,b^ ± 0.01	0.15^a,b^ ± 0.02	0.09^a^ ± 0.02	0.0025
C18:2_CLA_4	0.33^c^ ± 0.05	0.00^a^ ± 0.00	1.12^b^ ± 0.19	0.00^a^ ± 0.00	1.39^d^ ± 0.08	0.19^a,b^ ± 0.06	0.0001
St/Lb	0.06^a^ ± 0.04	0.03^a^ ± 0.01	0.24^b^ ± 0.01	0.09^a^ ± 0.01	2.49^d^ ± 2.18	1.98^c^ ± 0.10	0.0134
O/V	0.44^c^ ± 0.01	0.01^a^ ± 0.01	1.45^d^ ± 0.02	0.03^a^ ± 0.01	7.11^e^ ± 1.13	0.12^b^ ± 0.2	0.0001
∑U	33.33^b^ ± 1.53	34.54^b^ ± 1.68	49.51^d^ ± 6.10	43.75^c^ ± 2.16	46.33^c^ ± 6.67	28.17^a^ ± 3.83	0.0002
∑S	66.66^b^ ± 4.14	65.44^b^ ± 1.57	50.50^a^ ± 5.79	56.26^a^ ± 1.92	53.66^a^ ± 5.34	71.84^c^ ± 5.44	0.0004
U/S	0.50^b^ ± 0.01	0.53^b^ ± 0.01	0.98^e^ ± 0.01	0.78^c^ ± 0.01	0.86^d^ ± 0.03	0.39^a^ ± 0.02	0.0001
∑CLA	1.39^b^ ± 0.21	0.51^a^ ± 0.09	3.59^c^ ± 0.59	0.44^a^ ± 0.05	4.24^d^ ± 0.44	0.81^a^ ± 0.18	0.0001

Legend: as Table 2.

^a,b,c,d,e,f^–Means with different lowercase letters the same line indicate a significant difference at the significance level of 0.05 (Tukey HSD).

We observed significant differences in the proportions of saturated, unsaturated, and cyclic fatty acids compared to previous studies (Rizzo et al., 1987; Kankaanpaa et al., 2004; Machado et al., 2004; Liong and Shah, 2005; Corsetti et al., 2010; Rodrigues et al., 2012). Notably, our findings revealed substantial levels of dihydrosterculic and lactobacillic acids, cyclic derivatives of oleic and vaccenic acids, respectively. These results contrast with some prior studies that may have missed these cyclic acids due to separation and detection challenges (Machado et al., 2004; Liong and Shah, 2005). For *L. casei*, our dominant fatty acids included dihydrosterculic and lactobacillic acids, alongside palmitic acid. This aligns with the findings of Rizzo et al. (1987) who reported palmitic, octadecenoic, and cyclopropane acids as the major components in *L. casei* ATCC 393. However, some discrepancies exist, such as the presence of palmitoleic acid in Machado et al. (2004) which we did not observe. These differences could be attributed to various factors, including strain variations and growth conditions. While comparisons for *L. paracasei* subsp. paracasei MB were limited due to a scarcity of data specifically for this subspecies, it’s important to acknowledge that the *L. casei* group taxonomy has undergone revisions in recent years. Some strains previously classified as L. casei may now be identified as *L. paracasei*. Future studies that incorporate a wider range of *Lactobacillus* species, including recently reclassified members of the casei group, along with standardized protocols, would offer valuable insights into the influence of growth media components on bacterial fatty acid composition.

### *Limosilactobacillus fermentum* and *Limosilactobacillus reuteri*

The fatty acid profile of bacterial biomass of *L. fermentum* ATCC 9338 and *L. reuteri* DSM 17938 cultured on growth medium with Tween 80™ was characterized by the presence of 27 and 28 fatty acids, respectively (Table 4). We demonstrated that *L. fermentum* ATCC 9338 was characterized by the share of palmitic (C16:0), oleic (C18:1,*cis*-9), lactobacillic (*cyc*C19:0,*cis*-10,11), *cis*-vaccenic (C18:1,*cis*-11), and dihydrosterculic fatty (*cyc*C19:0,*cis*-9,10) acids, while *L. reuteri* DSM 17938 by palmitic, *cis*-vaccenic (C18:1,*cis*-11), dihydrosterculic (*cyc*C19:0,*cis*-9,10), and lactobacillic (*cyc*C19:0,*cis*-10,11) acids. Each of these strains demonstrated the ability to synthesize vaccenic and lactobacillic (*cyc*C19:0,*cis*-10,11) acids, which were in a pool of dominant fatty acids. We also demonstrated the presence of oleic acid (C18:1,*cis*-9) and its cyclic derivative, dihydrosterculic acid (*cyc*C19:0,*cis*-9,10) (Table 4). *L. fermentum* ATCC 9338 and *L. reuteri* DSM 17938 showed excellent growth on MRS agar without Tween 80™. The fatty acid profile of the first of these strains contained 20 fatty acids, while the second strain was characterized by 22 fatty acids. Removal of Tween 80™ from the growth medium resulted in statistically significant changes in the fatty acid profile of bacterial strains: an increase in the share of 8 fatty acids (5 unsaturated fatty acids and 3 saturated fatty acids) and a decrease in the share of 11 fatty acids (4 saturated fatty acids and 7 unsaturated fatty acids). This significantly reduced the calculated ratios of O/V and U/S, but increased the calculated St/Lb ratio. It is also worth noting that the changes observed were not the same across all bacterial strains tested, which, as with the lactobacilli strains discussed previously, allows for some differentiation.

**TABLE 4:** The percentage fatty acids composition (mean ± SD, n = 6) in mesophilic *Limosilactobacillus fermentum* and *Limosilactobacillus reuteri* strains profiles obtained from the cell cultured in a growth medium with Tween 80^TM^ (with T80) and without Tween 80^TM^ (no T80).

Strain symbol	*L. fermentum* ATCC 9338		*L. reuteri* DSM 17938		*p-value*
Fatty acid/Medium	With T80	No T80	With T80	No T80	
C10:0	0.17^a^ ± 0.02	0.05^a^ ± 0.06	0.15^a^ ± 0.09	0.13^a^ ± 0.09	0.2550
C12:0	1.10^c^ ± 0.12	0.12^a^ ± 0.08	0.78^b^ ± 0.14	0.22^a^ ± 0.07	0.0001
C14:0	1.32^a^ ± 0.12	1.07^a^ ± 0.39	2.37^b^ ± 0.23	1.39^a^ ± 0.31	0.0022
15:0,*iso*	0.06^a^ ± 0.01	0.04^a^ ± 0.04	0.08^a^ ± 0.04	0.04^a^ ± 0.07	0.6702
15:0,*anteiso*	0.03^a^ ± 0.00	0.03^a^ ± 0.04	0.01^a^ ± 0.02	0.02^a^ ± 0.03	0.7707
C15:0	0.05^a^ ± 0.00	0.05^a^ ± 0.07	0.07^a^ ± 0.02	0.06^a^ ± 0.02	0.8983
C16:0	27.11^a^ ± 1.97	30.28^a^ ± 1.06	40.07^b^ ± 1.79	46.07^b^ ± 4.12	0.0001
C16:1,*trans-*9	1.11^a^ ± 0.03	2.00^b^ ± 0.10	1.94^a,b^ ± 0.21	3.07^c^ ± 0.30	0.0001
C16:1,*cis-*9	0.76^b^ ± 0.06	0.75^b^ ± 0.12	0.46^a^ ± 0.02	0.44^a^ ± 0.09	0.0515
C12:0,2OH	0.09^c^ ± 0.01	0.05^b^ ± 0.01	0.00^a^ ± 0.00	0.00^a^ ± 0.00	0.0001
*cyc*C17:0,*cis-*9,10	0.00^a^ ± 0.00	0.05^a^ ± 0.11	0.02^a^ ± 0.05	0.00^a^ ± 0.00	0.7185
C18:0	1.01^a^ ± 0.08	1.66^a^ ± 0.36	3.28^b^ ± 0.60	10.66^c^ ± 1.33	0.0001
C18:1	0.09^c^ ± 0.01	0.00^a^ ± 0.00	0.02^a,b^ ± 0.05	0.00^a^ ± 0.00	0.0074
C18:1,*trans-*6	0.11^a^ ± 0.01	0.26^b^ ± 0.02	0.10^a^ ± 0.02	0.24^b^ ± 0.05	0.0002
C18:1,*trans-*9	0.13^a^ ± 0.02	0.34^b,c^ ± 0.04	0.12^a^ ± 0.01	0.26^b^ ± 0.07	0.0004
C18:1,*trans-*11	0.34^a^ ± 0.09	0.00^a^ ± 0.00	0.34^a^ ± 0.83	0.00^a^ ± 0.00	0.5976
C18:1,*cis-*6	0.25^a^ ± 0.02	0.55^a,b^ ± 0.05	1.49^c^ ± 0.69	1.51^c^ ± 0.31	0.0066
C18:1,*cis-*9	18.41^d^ ± 1.35	0.47^a^ ± 0.17	9.07^c^ ± 0.45	1.09^b^ ± 1.01	0.0001
C18:1,*cis-*11	14.11^a,b^ ± 0.12	22.34^b^ ± 0.84	14.64^a,b^ ± 2.90	10.26^a^ ± 1.55	0.0002
C18:2,*trans-*9,*trans-*12	0.02^a^ ± 0.02	0.00^a^ ± 0.00	0.29^a^ ± 0.70	0.01^a^ ± 0.02	0.7046
C18:2,*cis-*9, *cis-*12	0.00^a^ ± 0.01	0.00^a^ ± 0.00	0.03^a^ ± 0.04	0.07^a^ ± 0.16	0.7029
*cyc*C19:0,*cis-*9,10	14.06^b^ ± 0.51	0.55^a^ ± 0.10	12.18^b^ ± 2.80	1.45^a^ ± 1.31	0.0001
*cyc*C19:0,*cis-*10,11	17.70^b^ ± 0.94	39.25^d^ ± 1.35	10.35^a^ ± 0.58	22.09^c^ ± 2.86	0.0001
18:2,*cis-*9,*trans-*11	0.64^b^ ± 0.05	0.08^a^ ± 0.01	0.68^b^ ± 0.05	0.75^b^ ± 0.11	0.0001
C18:2CLA_1	0.06^b^ ± 0.01	0.00^a^ ± 0.00	0.08^b^ ± 0.02	0.00^a^ ± 0.00	0.0001
18:2,*trans-*10,*cis-*12	0.67^c^ ± 0.06	0.00^a^ ± 0.00	0.43^b^ ± 0.06	0.00^a^ ± 0.00	0.0001
C18:2_CLA_2	0.05^b^ ± 0.01	0.00^a^ ± 0.00	0.06^b^ ± 0.02	0.00^a^ ± 0.00	0.0002
C18:2_CLA_3	0.04^a^ ± 0.02	0.00^a^ ± 0.00	0.01^a^ ± 0.02	0.04^a^ ± 0.05	0.2763
C18:2_CLA_4	0.52^c^ ± 0.05	0.00^a^ ± 0.00	0.90^d^ ± 0.04	0.12^a,b^ ± 0.13	0.0001
St/Lb	0.21^a^ ± 0.21	0.26^b^ ± 0.31	0.19^a^ ± 0.18	0.26^b^ ± 0.32	0.0001
O/V	0.92^b^ ± 0.54	0.09^a^ ± 0.11	0.92^b^ ± 0.42	0.06^a^ ± 0.07	0.0001
∑U	37.05^b^ ± 0.37	24.25^a^ ± 3.59	33.01^b^ ± 3.33	21.65^a^ ± 5.35	0.0016
∑S	69.21^a^ ± 9.21	82.77^b^ ± 13.53	64.14^a^ ± 7.38	75.83^b^ ± 8.91	0.0378
U/S	0.54^b^ ± 0.08	0.30^a^ ± 0.09	0.52^b^ ± 0.11	0.29^a^ ± 0.10	0.0001
∑CLA	2.17^b^ ± 0.28	0.64^a^ ± 0.79	1.97^b^ ± 0.27	0.49^a^ ± 0.59	0.0001

Legend: as Table 2.

^a,b,c,d^–Means with different lowercase letters the same line indicate a significant difference at the significance level of 0.05 (Tukey HSD).

Our study confirmed previous findings (Johnsson et al., 1995) that Tween 80™ significantly alters the fatty acid profile of *L. fermentum*. Oleic (C18:1,*cis*-9) and dihydrosterculic (*cyc*C19:0,*cis*-9,10) acids, undetectable without the surfactant, became enriched upon its addition. This effect occurred without affecting growth rate, suggesting independent processes. Furthermore, our results aligned with Rizzo et al. (1987) regarding the overall fatty acid composition of *L. fermentum*, highlighting the consistency of this profile across strains.

Taranto et al. (2006) determined the dominant fatty acid in *L. reuteri* CRL 1098 to be palmitic acid (57.7%). Our study found a similar proportion of palmitic acid (40.07%) in *L. reuteri* cells, suggesting a relatively consistent fatty acid profile for this species. Similar results were observed for stearic acid (3.86% and 3.28%, respectively) and the ratio of oleic acid (C18:1,*cis*-9) to *cis*-vaccenic acid (C18:1,*cis*-11) ratio (0.70% and 0.62%). Comparison of cyclic acids was difficult due to limitations in the cited study (Taranto et al., 2006). The ability of *L. reuteri* strains to synthesize CLA from linoleic acid (C18:2,*cis*-9,*cis*-12) has been demonstrated previously (Jenkins and Courtney, 2003; Hernandez-Mendoza et al., 2009; Macouzet et al., 2010). Our study confirmed CLA synthesis by *L. reuteri* DSM 17938 at a level comparable to other strains. The dominant CLA profile was *cis-*9,*trans-*11-octadecadienoic acid, and the strain exhibited the ability to synthesize CLA even without added oleic acid. Lactic acid bacteria possess the ability to transform unsaturated fatty acids in their conjugated form (i.e., CLA) (Kishino et al., 2002; Lin et al., 2002; Lin et al., 2003; Ogawa et al., 2005; Lin, 2006).

### Lactiplantibacillus plantarum

Our study examined three strains of *L. plantarum*, all of which exhibited efficient growth on MRS agar with or without Tween 80™. Interestingly, the fatty acid profiles of *L. plantarum* 299v and *L. plantarum* B.01149cultured on growth medium with Tween 80™ exhibiting profiles with 24 fatty acids, and B.01834 exhibiting a 24-fatty acid profile. Cultivation on growth medium with the addition of Tween 80™ resulted in the fatty acid profile of all three strains examined being dominated by palmitic acid (C16:0), dihydrosterculic acid, *cis*-vaccinic acid and lactobacillic acid (*cyc*C19:0,*cis*-10,11), with the involvement of the last two of these fatty acids differentiating the strains examined (Table 5). Removal of Tween 80™ from the growth medium resulted in a decrease in the share of 11 fatty acids (4 saturated and 7 unsaturated) in the fatty acid profile and an increase in the share of 9 fatty acids (6 saturated and 3 unsaturated) in the fatty acid profile. It is also worth mentioning that the overall contribution of CLA to the fatty acid profile of the tested strains was also statistically reduced due to the removal of Tween 80™ from the growth medium. As a result, the proportion of total saturated and unsaturated fatty acids in the fatty acid profile increased significantly in some bacterial strains. Instead, a significant increase in the St/Lb ratio and a significant decrease in the O/V ratio were observed.

**TABLE 5:** The percentage fatty acids composition (mean ± SD, n = 6) in mesophilic *Lactiplantibacillus plantarum* strains profiles obtained from the cell cultured in a growth medium with Tween 80^TM^ (with T80) and without Tween 80^TM^ (no T80).

Strain symbol	*L. plantarum* 299v		*L. plantarum* B.01149		*L. plantarum* B.01834		*p-value*
Fatty acid/Medium	With T80	No T80	With T80	No T80	With T80	No T80	
C10:0	0.21^a^ ± 0.08	0.31^a,b^ ± 0.09	0.29^a^ ± 0.12	0.46^b,c^ ± 0.07	0.38^b^ ± 0.07	0.52^c^ ± 0.04	0.0057
C12:0	0.90^a,b^ ± 0.21	0.62^a,b^ ± 0.19	0.98^b^ ± 0.50	1.00^b,c^ ± 0.19	1.91^c^ ± 0.65	0.97^b^ ± 0.15	0.0185
C14:0	2.79^b^ ± 0.35	1.45^a^ ± 0.35	4.21^c^ ± 0.27	2.04^a,b^ ± 0.21	6.16^d^ ± 1.92	2.70^b^ ± 0.46	0.0002
15:0,*iso*	0.12^a^ ± 0.02	0.27^b^ ± 0.05	0.18^a^ ± 0.15	0.49^b^ ± 0.05	0.27^b^ ± 0.06	0.45^c^ ± 0.05	0.0003
15:0,*anteiso*	0.05^a^ ± 0.06	0.00^a^ ± 0.00	0.04^a^ ± 0.05	0.02^a^ ± 0.05	0.18^a^ ± 0.17	0.02^a^ ± 0.04	0.1576
C15:0	0.05^a,b^ ± 0.06	0.01^a^ ± 0.03	0.05^a,b^ ± 0.06	0.02^a^ ± 0.05	0.17^b^ ± 0.10	0.01^a^ ± 0.02	0.0472
C16:0	44.99^a,b^ ± 2.37	52.95^c^ ± 1.73	41.79^a^ ± 2.24	54.78^d^ ± 0.87	40.11^a^ ± 2.34	52.82^c^ ± 0.96	0.0001
C16:1,*trans-*9	0.90^a^ ± 0.07	1.21^b^ ± 0.06	1.07^a,b^ ± 0.05	1.23^b^ ± 0.10	1.03^a,b^ ± 0.07	1.40^c^ ± 0.14	0.0002
C16:1,*cis-*9	2.31^b^ ± 0.26	1.30^a^ ± 0.09	2.76^b^ ± 0.45	1.27^a^ ± 0.14	2.73^b^ ± 0.51	1.44^a^ ± 0.22	0.0001
C12:0,2OH	0.08^a^ ± 0.04	0.10^a^ ± 0.01	0.12^a^ ± 0.06	0.11^a^ ± 0.01	0.03^a^ ± 0.07	0.07^a^ ± 0.08	0.3858
*cyc*C17:0,*cis-*9,10	0.27^b^ ± 0.16	0.37^c^ ± 0.04	0.16^a^ ± 0.03	0.37^c^ ± 0.03	0.13^a^ ± 0.02	0.31^b,c^ ± 0.04	0.0046
C18:0	1.11^c^ ± 0.43	3.23^b^ ± 0.26	1.22^a^ ± 0.10	4.12^b^ ± 0.36	1.39^a^ ± 0.17	3.58^b^ ± 0.44	0.0001
C18:1	0.15^b^ ± 0.04	0.00^a^ ± 0.00	0.13^b^ ± 0.06	0.00^a^ ± 0.01	0.12^b^ ± 0.02	0.00^a^ ± 0.00	0.0001
C18:1,*trans-*6	0.14^a^ ± 0.02	0.11^a^ ± 0.03	0.07^a^ ± 0.05	0.11^a^ ± 0.02	0.10^a^ ± 0.02	0.13^a^ ± 0.01	0.1104
C18:1,*trans-*9	0.16^a^ ± 0.03	0.20^a^ ± 0.09	0.11^a^ ± 0.05	0.16^a^ ± 0.02	0.13^a^ ± 0.03	0.17^a^ ± 0.01	0.2983
C18:1,*trans-*11	0.08^a,b^ ± 0.02	0.00^a^ ± 0.00	0.16^c^ ± 0.06	0.01^a^ ± 0.03	0.12^b^ ± 0.06	0.00^a^ ± 0.00	0.0006
C18:1,*cis-*6	0.14^a^ ± 0.03	0.29^a,b^ ± 0.03	0.21^a,b^ ± 0.07	0.35^b^ ± 0.03	0.19^a^ ± 0.07	0.29^a,b^ ± 0.03	0.0015
C18:1,*cis-*9	4.03^c^ ± 0.45	0.46^a,b^ ± 0.34	4.18^c^ ± 0.52	0.11^a^ ± 0.03	4.04^c^ ± 0.45	0.15^a^ ± 0.26	0.0001
C18:1,*cis-*11	12.09^a^ ± 0.49	22.39^b^ ± 1.60	12.96^a^ ± 1.16	20.06^b^ ± 0.56	12.03^a^ ± 2.06	19.89^b^ ± 0.85	0.0001
C18:2,*trans-*9,*trans-*12	0.00^a^ ± 0.00	0.00^a^ ± 0.00	0.00^a^ ± 0.00	0.01^a^ ± 0.02	0.00^a^ ± 0.00	0.00^a^ ± 0.00	0.6017
C18:2,*cis-*9, *cis-*12	0.06^a^ ± 0.04	0.03^a^ ± 0.07	0.00^a^ ± 0.00	0.00^a^ ± 0.00	0.00^a^ ± 0.00	0.00^a^ ± 0.00	0.1991
*cyc*C19:0,*cis-*9,10	14.76^b^ ± 1.63	0.15^a^ ± 0.15	16.28^b^ ± 2.14	0.28^a^ ± 0.06	14.91^b^ ± 1.39	0.46^a^ ± 0.31	0.0001
*cyc*C19:0,*cis-*10,11	12.95^a,b^ ± 0.62	14.39^b,c^ ± 0.53	11.02^a^ ± 0.96	12.82^a,b^ ± 0.55	11.69^a^ ± 2.04	14.46^b,c^ ± 0.91	0.0101
18:2,*cis-*9,*trans-*11	0.55^b^ ± 0.07	0.16^a^ ± 0.03	0.83^b^ ± 0.20	0.18^a^ ± 0.02	0.83^c^ ± 0.11	0.19^a^ ± 0.05	0.0001
C18:2CLA_1	0.00^a^ ± 0.00	0.00^a^ ± 0.00	0.00^a^ ± 0.00	0.00^a^ ± 0.00	0.00^a^ ± 0.00	0.00^a^ ± 0.00	0.8421
18:2,*trans-*10,*cis-*12	0.63^b^ ± 0.08	0.00^a^ ± 0.00	0.67^b^ ± 0.14	0.00^a^ ± 0.00	0.73^b^ ± 0.11	0.00^a^ ± 0.00	0.0001
C18:2_CLA_2	0.00^a^ ± 0.00	0.00^a^ ± 0.00	0.00^a^ ± 0.00	0.00^a^ ± 0.00	0.15^a^ ± 0.22	0.00^a^ ± 0.00	0.2939
C18:2_CLA_3	0.00^a^ ± 0.00	0.00^a^ ± 0.00	0.00^a^ ± 0.00	0.00^a^ ± 0.00	0.00^a^ ± 0.00	0.00^a^ ± 0.00	0.5747
C18:2_CLA_4	0.50^b^ ± 0.07	0.00^a^ ± 0.00	0.52^b^ ± 0.13	0.00^a^ ± 0.00	0.50^b^ ± 0.09	0.00^a^ ± 0.00	0.0001
St/Lb	0.09^a^ ± 0.03	0.22^b^ ± 0.01	0.11^a^ ± 0.01	0.32^b^ ± 0.01	0.12^a^ ± 0.01	0.25^b^ ± 0.01	0.0001
O/V	0.33^b^ ± 0.02	0.02^a^ ± 0.01	0.32^b^ ± 0.01	0.01^a^ ± 0.01	0.34^b^ ± 0.02	0.01^a^ ± 0.01	0.0001
∑U	21.74^a^ ± 1.67	26.15^a^ ± 2.34	23.67^a^ ± 2.94	23.49^a^ ± 0.98	22.70^a^ ± 3.82	23.66^a^ ± 1.57	0.4042
∑S	78.28^a^ ± 6.03	73.85^a^ ± 3.43	76.34^a^ ± 6.68	76.51^a^ ± 2.50	77.33^a^ ± 9.00	76.37^a^ ± 3.50	0.9544
U/S	0.28^a^ ± 0.01	0.36^b^ ± 0.02	0.31^a,b^ ± 0.01	0.31^a,b^ ± 0.01	0.29^a^ ± 0.02	0.31^a,b^ ± 0.01	0.0001
∑CLA	1.68^b^ ± 0.22	0.16^a^ ± 0.03	2.02^c^ ± 0.47	0.18^a^ ± 0.02	2.21^c^ ± 0.53	0.19^a^ ± 0.05	0.0001

Legend: as Table 2.

^a,b,c,d^–Means with different lowercase letters the same line indicate a significant difference at the significance level of 0.05 (Tukey HSD).

We identified discrepancies with Johnsson et al. (1995) regarding oleic acid (C18:1,*cis*-9) and lactobacillic acid (*cyc*C19:0,*cis*-10,11) production by *L. plantarum* 2004. Our strains displayed a dominance of lactobacillic acid (11.02%–14.46%) and lower levels of oleic acid (up to 4.18%) compared to their findings. We attribute these differences to potential limitations in their methodology, such as sample size and incubation temperature. Our results for strains cultured without Tween 80™ showed a high proportion of palmitic acid (52.82%–54.78%), aligning with Rozès and Peres (1998). Both studies highlighted the prominence of C18:1 and cyclic C19:0 acids, although the specific compositions were not identical. Similarly, Russell et al. (1995) reported palmitic acid dominance, but stearic acid (C18:0) was not a major component in our analysis. These variations likely stem from differing incubation temperatures and analytical methods. Rizzo et al. (1987) identified palmitic (C16:0), cis-vaccenic (C18:1,cis-11), and cyclic C19:0 acids in *L. plantarum* ATCC 14917. However, their classification of the latter solely as lactobacillic acid (cycC19:0,cis-10,11) might not account for all possible isomers. *L. plantarum*’s ability to produce dihydrosterculic acid (cycC19:0,cis-9,10), as demonstrated by Johnsson et al. (1995), supports this notion. Our findings support this notion, revealing the presence of both lactobacillic (*cyc*C19:0,*cis*-10,11) and dihydrosterculic (*cyc*C19:0,*cis*-9,10) acids. Our research confirms the ability of *L. plantarum* strains to synthesize CLA (conjugated linoleic acid) from various substrates, as reported in previous studies (Ogawa et al., 2005; Kishino et al., 2009; Corsetti et al., 2010; Rodríguez-Alcalá et al., 2011). Studies by Kishino et al. (2009) CLA levels were significantly higher in cultures supplemented with Tween 80™ (1.67%–2.21%) compared to those without (0.16%–0.19%). The two predominant CLA isomers identified (cis-9,trans-11 and trans-10,cis-12) are consistent with Rodriguez-Alcalá et al. (2011). This study highlights the variability in fatty acid profiles of *L. plantarum* strains and emphasizes the need for standardized methodologies for accurate comparisons. We confirm the prevalence of palmitic (C16:0) and C18:1/cyclic C19:0 fatty acids, with lactobacillic acid (*cyc*C19:0,*cis*-10,11) as the dominant cyclic form. Additionally, our findings support *L. plantarum*’s ability to produce CLA, with levels influenced by the presence of Tween 80™.

### Principal components analysis

**FIGURE 1:**
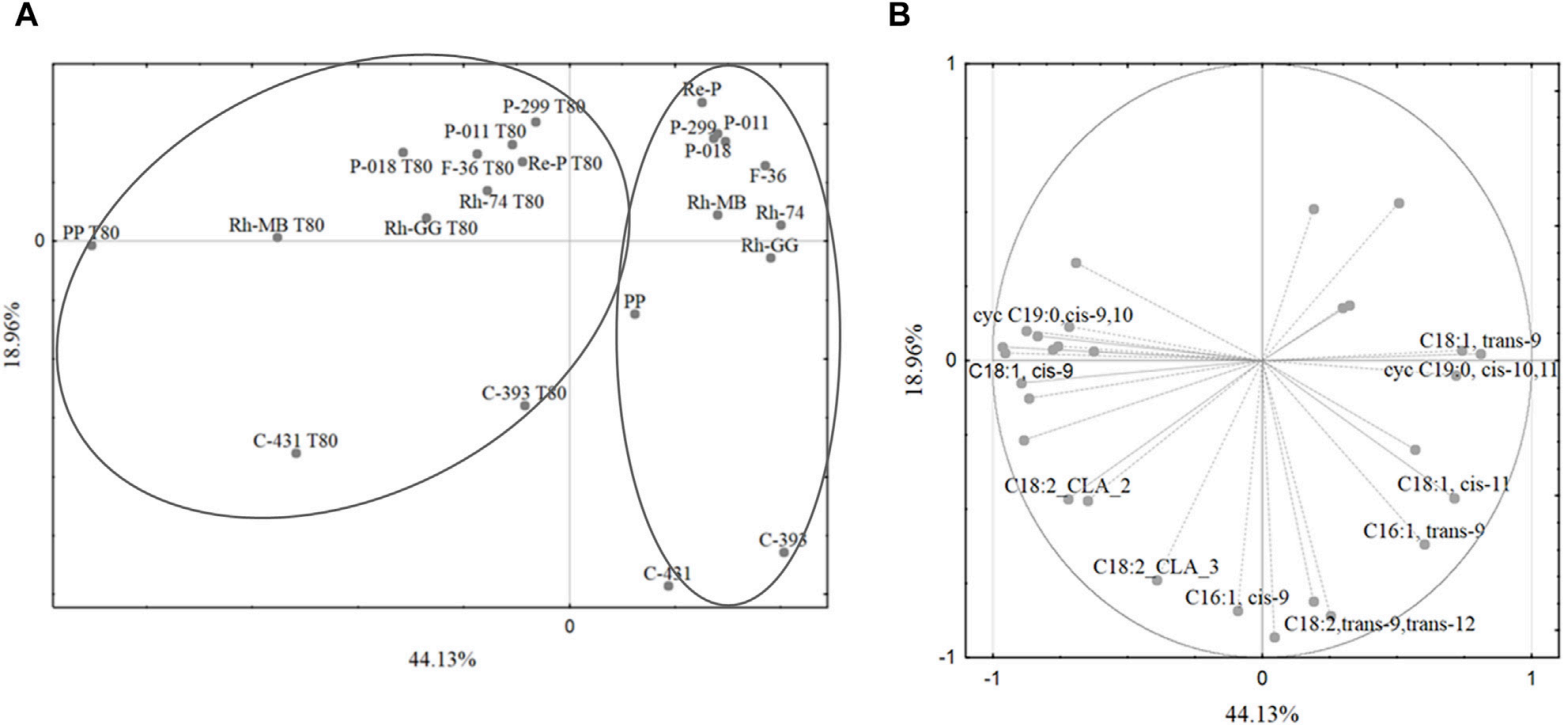
**(A)** The grouping of cases based on the shares of fatty acids in the mesophilic lactobacilli biomass. The projection of cases onto SG-1 (44.13%) and SG-2 (18.96%) plane. **(B)** The projection of variables (composition and shares of acid groups) onto SG-1 (44.13%) and SG-2 (18.96%) plane. The differentiation of mesophilic lactobacilli based on the fatty acids composition. Legend: as Table 2.

Fatty acids play a crucial role in the physiology and metabolism of lactobacilli. Their composition can influence various aspects of lactobacilli growth, function, and interaction with the environment. Understanding the fatty acid profiles of lactobacilli can provide valuable insights into their metabolic capabilities and potential applications. Of course, the fatty acid composition of lactobacilli can vary significantly depending on the strain and growth conditions. Principal components analysis revealed that growth medium supplementation with Tween 80™ significantly affected the fatty acid profiles of mesophilic lactobacilli strains, particularly the share of oleic acid (C18:1,*cis*-9), *cis-*vaccenic acid (C18:1,*cis*-11), lactobacillic acid (*cyc*C19:0,*cis*-10,11), and dihydrosterculic acid (*cyc*C19:0,*cis*-9,10). In general, the values of the first two principal components of the PCA statistical analysis of 57.53% are not too high and indicate that they do not fully explain the variability of the original data set. Nevertheless, the first two principal components may be statistically significant for the variables we are interested in. The PCA gives a quick overview of the connection between the different factors and the samples within. It should be noted that the first main component, which is 57.53% of the model variation, divided the cases into two main groups: the fatty acid profiles obtained from the growth medium with Tween 80™ and without Tween 80™ (Figure 1A). The first principal component separated the cases into two main groups based on the growth medium with or without Tween 80™ (Figure 1A). Notably, some *L. casei* strains did not cluster together, suggesting a potential taxonomic misclassification or high variability within this group. The high share of oleic acid (C18:1,*cis*-9) and CLA was important for the separation of the bacteria cultured on the growth medium with Tween 80™, while linoelaidic (C18:2,*trans*-9,*trans*-12), palmitelaidic (C16:1,*trans*-9), and *cis-*vaccenic (C18:1,*cis*-11) acids were important in case of growth medium without Tween 80™ (Figure 1B). This allowed for the separation of strains cultured on growth medium with Tween 80™ into two main groups: those cultured with Tween 80™ and cultured without Tween 80™. In contrast, no significant differentiation was observed for strains grown on the same growth medium without Tween 80™.

Recent taxonomic revisions suggest that some *L. casei* strains might be reclassified as *L. paracasei* (Ghosh et al., 2019). Unfortunately, we could not perform phylogenetic analysis on all our strains due to the lack of a genome sequence for C-431. Despite this uncertainty, we observed distinct fatty acid profiles among the three *L. casei* strains (potentially *L. paracasei*). However, further studies with a larger and well-characterized strain collection are necessary to confirm the discriminatory power of fatty acid profiles for differentiation between these closely related species.

The exact effects of the presence of Tween 80™ in the growth medium vary depending on the type of lactobacilli, but in general, Tween 80™ can increase saturated fatty acids and reduce unsaturated fatty acids. The presence of Tween 80™ in the growth medium generally increased the proportion of lactobacillic acid (*cyc*C19:0,*cis*-10,11) in the fatty acid profile of all lactobacilli strains. This was due to the stimulation of the synthesis of lactobacillic acid (*cyc*C19:0,*cis*-10,11) by lactobacilli cells in response to the absence of oleic acid (C18:1,*cis*-9) from Tween 80™. The presence of Tween 80™ in the growth medium had a mixed effect on the proportion of myristic acid (C14:0) and dihydrosterculic acid (*cyc*C19:0,*cis*-9,10) in the fatty acid profile. In some lactobacilli strains, the proportion of myristic acid and dihydrosterculic acid (*cyc*C19:0,*cis*-9,10) increased, while in other species, the proportion of myristic acid and dihydrosterculic acid (*cyc*C19:0,*cis*-9,10) decreased. The presence of Tween 80™ in the growth medium significantly impacts the fatty acid profile of lactobacilli, generally increased the proportion of palmitic acid in the fatty acid profile of all lactobacilli strains. This was due to the incorporation of oleic acid (C18:1,*cis*-9) from Tween 80™ into the cell membranes of lactobacilli cells, which increased the demand for saturated fatty acids to maintain the fluidity of the cell membranes. While our data suggests a decrease in *cis*-vaccenic acid (C18:1,*cis*-11) in the fatty acid profile of all lactobacilli strains, further investigation is needed to elucidate the underlying mechanisms. Existing literature suggests that Tween 80™ might suppress early fatty acids synthesis, potentially affecting *cis*-vaccenic acid (C18:1,*cis*-11) production later in the growth cycle (Jacques et al., 1980; Zotta et al., 2017b; Reitermayer et al., 2018). Future studies employing time-course experiments could provide valuable insights into the dynamics of fatty acid biosynthesis and incorporation in the presence of Tween 80™, allowing for a more definitive understanding of potential competition or alternative pathways involved.

## Conclusion

Our study investigated the influence of Tween 80™, a widely used surfactant in culturing media, on the fatty acid profile of selected mesophilic lactobacilli strains. The fatty acid composition of mesophilic lactobacilli cells plays a crucial role in determining their membrane properties, which are essential for various cellular functions, including cell growth, motility, and stress resistance. Our study investigated the impact of Tween 80™, a common surfactant used in bacterial culturing media, on the fatty acid profile of selected mesophilic lactobacilli strains. Tween 80™ has a significant impact on the fatty acid profile of lactobacilli. The presence of Tween 80™ in the growth medium can alter the proportion of various fatty acids, including saturated and unsaturated. The specific changes in fatty acid composition vary depending on the lactobacilli strains and species. One important aspect to consider is the role of the culturing environment in shaping the fatty acid profile of lactobacilli. Culture media can introduce external fatty acids, such as the oleic acid (C18:1,*cis*-9) present in Tween 80™, a common additive. Notably, oleic acid originating in Tween 80™ can be incorporated into lactobacilli membranes, contributing to the complex interplay between environmental factors and bacterial fatty acid biosynthesis in shaping membrane properties.

Our results reveal that the presence of Tween 80™ in the growth medium decreases the abundance of *cis*-vaccenic acid (C18:1,*cis*-11), a key contributor to membrane fluidity. This suggests that Tween 80™ may not only act as an external fatty acid source but also potentially compete with the bacteria’s own synthesis of *cis*-vaccenic acid (C18:1,*cis*-11). While competition with oleic acid (C18:1,*cis*-9) from Tween 80™ for membrane incorporation is a possibility, further investigation is needed. Existing literature suggests that Tween 80™ may initially suppress fatty acid synthesis, potentially impacting the availability of precursors for later *cis*-vaccenic acid (C18:1,*cis*-11) production. This decrease in *cis*-vaccenic acid (C18:1,*cis*-11) may lead to an increase in membrane rigidity, which could negatively impact cell growth, motility, and stress resistance.

Conversely, the addition of Tween 80™ to the growth medium had the opposite effect, increasing the proportion of *cis*-vaccenic acid (C18:1,*cis*-11), likely through precursor availability. This increase may help to maintain membrane fluidity, which is important for these cellular functions. The presence of Tween 80™ can increase the proportion of palmitic acid (C16:0) and lactobacillic acid (*cyc*C19:0,*cis*-10,11). Palmitic acid is a saturated fatty acid that is involved in maintaining cell membrane structure and function. Lactobacillic acid (*cyc*C19:0,*cis*-10,11) is a cyclopropane fatty acid that is unique to lactobacilli and is thought to play a role in their interactions with other organisms.

In conclusion, our study demonstrates that Tween 80™ significantly impact the fatty acid composition of lactobacilli. The presence of Tween 80™ can reduce the proportion of oleic acid and *cis*-vaccenic acid (C18:1,*cis*-11). Oleic acid is a major component of the cell membranes of lactobacilli, and Tween 80™ can compete with oleic acid for incorporation into the membranes. The results of our study are particularly relevant to the industrial application of mesophilic lactobacilli. These changes in fatty acid composition can in turn lead to changes in membrane properties, which could have implications for cell function.

Future studies could explore the specific mechanisms by which Tween 80™ influences fatty acid synthesis and the potential for strain-specific adaptations. Additionally, investigating the functional consequences of altered membrane fluidity on cell function would provide valuable insights for optimizing probiotic performance.

## Data Availability

The original contributions presented in the study are included in the article/Supplementary Material, further inquiries can be directed to the corresponding author.
