# Supplementary material for: Tween 80™-induced changes in fatty acid profile of selected mesophilic lactobacilli
Source: Acta Biochim Pol. 2024 Jul 4;71:13014. doi: 10.3389/abp.2024.13014 (PMC11254618; doi:10.3389/abp.2024.13014)
Supplement: Supplementary file 1 [file Table1.docx]

Supplementary Material

Tween 80™-Induced Changes in Fatty Acid Profile of Selected Mesophilic Lactobacilli

Dorota Zaręba, Małgorzata Ziarno *

*** Correspondence:** Małgorzata Ziarno, malgorzata_ziarno@sggw.edu.pl

# Supplementary Tables

**Table 1S. Composition of the external standard (BAME) and identification parameters for each acid.**

| **Fatty acid** | **Acid name** | **tR (min)** | **ECL** | **EI** |
| --- | --- | --- | --- | --- |
| C11:0 | undecanoic | 15.466 | 11.000 | 74,87,143,157,200 |
| C12:0 | lauric / dodecanoic | 17.655 | 12.000 | 74,87,143,214 |
| C13:0 | tridecanoic | 19.751 | 13.000 | 74,87,143,185,228 |
| C14:0 | myristic / tetradecanoic | 21.757 | 14.000 | 74,87,143,199,242 |
| C10:0,2OH | 2-hydroxydecanoic | 22.658 | 14.481 | 69,83,143,228 |
| C15:0,iso | iso-13-methyltetradecanoic | 22.777 | 14.542 | 74,87,143,213,256 |
| C15:0,anteiso | anteiso-12-methyltetradecanoic | 23.084 | 14.701 | 74,87,143,213,256 |
| C15:0 | pentadecanoic | 23.673 | 15.000 | 74,87,143,213,256 |
| C16:0,iso | iso-14-methylpentadecanoic | 24.647 | 15.541 | 74,87,143,227,270 |
| C16:0 | palmitic / hexadecanoic | 25.505 | 16.000 | 74,87,143,227,270 |
| C17:0,iso | iso-15-methylhexadecanoic | 26.442 | 16.541 | 74,87,143,241,284 |
| C16:1,cis-9 | palmitoleic / hexadecenoic | 26.485 | 16.565 | 69,83,96,152,236 |
| C12:0,2OH | 2-hydroxydodecanoic | 26.636 | 16.653 | 69,83,97,171,230 |
| C17:0 | heptadecanoic | 27.255 | 17.000 | 74,87,143,241,284 |
| cycC17:0,cis-9,10 | cis-9,10-methylenehexadecanoic | 27.965 | 17.432 | 69,74, 83,97,250 |
| C18:0 | stearic / octadecanoic | 28.934 | 18.000 | 74,87,143,255,298 |
| C12:0,3OH | 3-hydroxydodecanoic | 29.175 | 18.159 | 71,74,83,103 |
| C18:1,trans-9 | elaidic / octadecenoic | 29.492 | 18.351 | 69,74,83,97,123,264 |
| C18:1,cis-9 | oleic / octadecenoic | 29.705 | 18.486 | 69,74,83,97,123,264 |
| C14:0,2OH | 2-hydroxytetradecanoic | 30.221 | 18.797 | 69,83,97,199 |
| C19:0 | nonadecanoic | 30.545 | 19.000 | 74,87,143,312 |
| C18:2,cis-9, cis-12 | linoleic / cis-9,cis12-octadecadienoic | 31.015 | 19.307 | 97,81,95,123,294 |
| cycC19:0,cis-9,10 | dihydrosterculic / cis-9,10-methylene-octadecanoic | 31.122 | 19.376 | 69,74,83,97,123,278 |
| C20:0 | eicosanic | 32.086 | 20.000 | 74,87,143,326 |
| C14:0,3-OH | 3-hydroxytetradecanoic | 32.592 | 20.318 | 71,74,103 |
| C16:0,2-OH | 2-hydroxyhexadecanoic | 33.475 | 20.864 | 69,83,97,227 |

Legend: EI - Electron Ionisation, tR – Retention Time
